# Comparison of Three Multiple Allergen Simultaneous Tests: RIDA Allergy Screen, MAST Optigen, and Polycheck Allergy

**DOI:** 10.1155/2013/340513

**Published:** 2012-12-24

**Authors:** Minje Han, Sue Shin, Hyewon Park, Kyoung Un Park, Myoung Hee Park, Eun Young Song

**Affiliations:** Department of Laboratory Medicine, Seoul National University College of Medicine and Seoul National University Hospital, Seoul 110-744, Republic of Korea

## Abstract

We compared the performances of 3 Multiple Allergen Simultaneous Test (MAST) assays: RIDA Allergy Screen (R-Biopharm, Darmstadt, Germany), MAST Optigen allergy system (Hitachi Chemical Diagnostics, Mountain View, CA), and Polycheck Allergy (Biocheck GmbH, Munster, Germany). Forty sera that tested positive with the RIDA Allergy Screen (20 for food and 20 for inhalant panel) were subjected to MAST Optigen and Polycheck Allergy. For 26 available sera with discrepant results, 62 ImmunoCAP allergen-specific IgE tests (Pharmacia Diagnostics, Uppsala, Sweden) were performed. Percent agreements (kappa value) were 87.6% (0.59) and 91.3% (0.60) between RIDA and MAST; 89.9% (0.55) and 88.3% (0.46) between RIDA and Polycheck; and 86.8% (0.51) and 90.6% (0.61) between MAST and Polycheck. Compared with ImmunoCAP, agreements (kappa value) of inhalant and food panels were 51.7% (0.04) and 33.3% (−0.38) for RIDA; 60.7% (0.27) and 81.8% (0.59) for MAST; and 65.5% (0.26) and 45.5% (0.07) for Polycheck. The agreements between RIDA, MAST, and Polycheck and ImmunoCAP-positivity were 45.7%, 88.2%, and 28.6%, respectively, and the agreements for ImmunoCAP-negativity were 37.0%, 51.9%, and 88.9%. MAST Optigen showed better agreement with ImmunoCAP than other assays in the food panel. Better sensitivity of MAST Optigen and better specificity of Polycheck Allergy were suspected.

## 1. Introduction

For the diagnosis of allergy, presence of allergen-specific immunoglobulin E (IgE) is usually established either by in vivo allergen skin tests or by in vitro allergen-specific IgE measurements [1, 2]. Although, in vivo skin test has been widely used to detect allergen-specific IgE. It is not a quantitative test and is difficult to be standardized [3]. Therefore, detection of allergen-specific IgE is important for the diagnosis of allergy [1, 2]. Since the development and improvement of fluorescent enzyme immunoassay, the ImmunoCAP system (Pharmacia Diagnostics AB, Uppsala, Sweden) has been widely accepted as a reference method of allergen-specific IgE measurement because of its reliability, reproducibility, and good accordance with allergen skin test. However, individual ImmunoCAP test can only detect IgE against a single allergen, making it quite expensive to use in a clinical setting [4].

Therefore, several multiple allergen simultaneous tests (MAST) were developed, which can detect more than 30 allergen-specific IgE [5–8]. However, allergen-specific IgE assays are often modified as manufacturers improve allergens or change reagents to optimize test performance, affecting the diagnostic performance of those assays. MAST Optigen (Hitachi Chemical Diagnostics, Mountain View, CA, USA), upgraded version of MAST CLA (Hitachi Chemical Diagnostics, Mountain View, CA, USA), and Polycheck Allergy (Biocheck GmbH, Munster, Germany) were recently introduced with good performances [6, 9, 10]. However, to the best of our knowledge, comparison of performances of those assays and analysis of concordance with ImmunoCAP system has not been performed. The aim of this study was to compare the performance of 3 MAST assays: RIDA Allergy Screen (R-Biopharm, Darmstadt, Germany), MAST Optigen allergy system (Hitachi Chemical Diagnostics, Mountain View, CA, USA), and Polycheck Allergy (Biocheck GmbH, Munster, Germany) compared to ImmunoCAP system as a reference method.

## 2. Material and Methods

### 2.1. Patients

Forty sera that tested positive with the RIDA Allergy Screen (20 for the food panel and 20 for the inhalant panel) in immunology laboratory of Seoul National University Hospital from October to December 2010 were stored in −70°C until thawing for MAST Optigen and Polycheck Allergy assays. Allergens and classifications of results of three MAST assays are summarized in Table 1. Specific IgE assay with ImmunoCAP FEIA system (Phadia, Uppsala, Sweden) as a reference method was performed on 26 available residual sera out of 32 sera showing discrepant results (0 class in one assay and ≥2 class in another assay) among three MAST assays. The Institutional Review Board of Seoul National University Hospital approved this study (IRB no. 1-2011-0038).

### 2.2. RIDA Allergy Screen

Two hundred and fifty *u*L of patient serum were added to reaction wells of each of inhalant and food panels which contain 39 kinds of allergens. After 45 min of incubation at room temperature and wash, 250 *u*L of Biotin tagged anti-IgE were added. After 45 minutes of incubation at room temperature and wash, 250 *u*L of streptavidin conjugate were added. Twenty minutes of incubation at room temperature and wash, 250 *u*L of luminescent reagent were added. After 20 minutes of incubation, results were scanned with CCD camera (RIDA X-Screen Reader) and interpreted as class 0–6. Class ≥1 was interpreted as positive.

### 2.3. Polycheck Allergy

After washing of inhalant and food cassette which contain 39 kinds of allergens, 250 *u*L of start solution were added. After 60 seconds of incubation, 200 *u*L of patient sera were added. After 1 hour of incubation on shaker, 6 times of washes were performed. Anti-IgE was added and 45 minutes of incubation on shaker was performed. After 3 times of washes, 250 *u*L of enzyme tagged conjugate were added. After 20 minutes of incubation and washes, 250 *u*L of luminescent reagent were added. After 20 minutes of incubation, results were scanned and interpreted with Biocheck Image Software as class 0–6. Class ≥1 was interpreted as positive.

### 2.4. MAST Optigen

Patient sera were added to MASTpette chambers which contain 35 kinds of allergens. After 2 hours of incubation and washes, enzyme-tagged anti-IgE was added. After 2 hours of incubation and washes, luminescent reagent was added. After 10 minutes of incubation, results were interpreted as class 0–4 with MAST Optigen luminometer. Class ≥1 was interpreted as positive.

### 2.5. ImmunoCAP System Allergen-Specific IgE

All procedures were performed following the manufacture's instruction. The detection range of ImmunoCAP FEIA was 0.1 to 100 kU/L. The sIgE classification scales were as follows: class 0: under 0.35 kU/L, class 1: 0.35–0.7 kU/L, class 2: 0.7–3.5 kU/L, class 3: 3.5–17.5 kU/L, class 4: 17.5–50 kU/L, class 5: 50–100 kU/L, class 6: over 100 kU/L. Class ≥1 was interpreted as positive.

### 2.6. Statistical Analysis

Agreement of detection results (Cohen's kappa analysis) was analyzed. We assessed and categorized Kappa value as almost perfect (0.8–1.0), substantial (0.6–0.8), moderate (0.4–0.6), fair (0.2–0.4), and poor (below 0.2) [11]. We calculated three different agreement percentages (positive, negative, and total agreement percentage). The positive and negative agreement percentages were calculated with the proportions of agreement for the average of their positive and negative responses. The total agreement percentage was calculated following: (total number of results − number of discrepancies) × 100/total number of results [12].

## 3. Results

For each of the MAST inhalant and food panels, percent agreements (kappa value) were 87.6% (0.59) and 91.3% (0.60) between RIDA Allergy Screen and MAST Optigen; 89.9% (0.55) and 88.3% (0.46) between RIDA Allergy Screen and Polycheck Allergy; and 86.8% (0.51) and 90.6% (0.61) between MAST Optigen and Polycheck Allergy (Table 2). 

Among the 20 sera tested by inhalant panel, for Housedust, most common inhalant allergen in Korean population [7], RIDA Allergy Screen was negative but MAST Optigen and Polycheck Allergy were positive on 4 sera (Table 3). Out of those 4 sera, 3 (with available residual sera) were tested by ImmunoCAP specific IgE. All of them showed positive results with ImmunoCAP (Table 4). Agreements of RIDA Allergy Screen, MAST Optigen, and Polycheck Allergy with ImmunoCAP-positive results for House dust were 0% (0/3), 100% (3/3), and 100% (3/3), respectively (Table 4).

For Peach, three MAST assays showed highest number of discrepant sera (On 5 sera, MAST Optigen Screen was positive but and RIDA Allergy Screen and Polycheck Allergy were negative.) (Table 3). Three of them, tested by ImmunoCAP specific IgE, showed positive results. Agreements of Allergy Screen, MAST Optigen, and Polycheck Allergy with ImmunoCAP-positive results for Peach were 0% (0/3), 100% (3/3), and 0% (0/3), respectively (Table 4). 

Compared with the 62 ImmunoCAP allergen-specific IgE test results for 26 discrepant sera, agreements (kappa value) of inhalant and food panels were 51.7% (0.04) and 33.3% (–0.38) for RIDA Allergy Screen; 60.7% (0.27) and 81.8% (0.59) for MAST Optigen; and 65.5% (0.26) and 45.5% (0.07) for Polycheck Allergy (Table 5). The agreements between RIDA Allergy Screen, MAST Optigen, and Polycheck Allergy results and ImmunoCAP-positive results were 53.8%, 91.7%, and 30.8% for inhalant panel; 40.9%, 86.4%, and 27.3% for food panel, respectively (Table 5). The agreements between RIDA Allergy Screen, MAST Optigen, and Polycheck Allergy results and ImmunoCAP-negative results were 50.0%, 37.5%, and 93.8% for inhalant panel; 18.2%, 72.7%, and 81.8% for food panel, respectively (Table 5).

## 4. Discussion

Although, most of evaluations of performance of MAST assays were performed compared to allergen skin test [6, 13–16], comparisons with ImmunoCAP assay have been performed [5, 8, 17] considering the limitation of allergen skin test as a reference method due to the difference of principle of in vivo test from in vitro test [1]. ImmunoCAP assay has been known to have established performance [2]. Our study was also performed compared to ImmunoCAP assay.

In this study, 3 MAST assays showed moderate agreement (86.8–91.3%, kappa 0.46–0.61) among them (Table 2). In comparison with ImmunoCAP, three MAST assays showed similar agreements for Inhalant panel (51.7–65.5%, kappa 0.04–0.27), and MAST Optigen showed better agreement (81.8%, kappa 0.59) than Polycheck Allergy (45.5%, kappa 0.07) or RIDA Allergy Screen (33.3%, kappa −0.38) for food panel (Table 4). In previous reports, the agreement between RIDA Allergy Screen and ImmunoCAP has been reported as 29.1% (kappa −0.303) on 633 discrepant sera between RIDA Allergy Screen and another MAST assay, AdvanSure system (LG Life Science, Seoul, Korea) [8]. Among 115 allergic patients, RIDA Allergy Screen showed 83.1% of agreement with ImmunoCAP for 10 common allergens [17]. Our result is similar to former one [8] because we also performed ImmunoCAP assays only on sera with discrepant results among three MAST assays.

 In our study, the agreement of MAST Optigen with ImmunoCAP-positive results was best (91.7% for inhalant panel and 86.4% for food panel) among 3 MAST assays (Table 5), implicating better sensitivity than other two assays. MAST CLA, previous version of MAST Optigen, has been reported to have slightly lower sensitivity (44.5%) than RIDA Allergy Screen (55.8%) or Polycheck Allergy (55.6%) [6]. The performance of MAST Optigen might be improved compared to MAST CLA as previous report [17]. 

The agreement of Polycheck Allergy with ImmunoCAP-negative results was best (93.8% for inhalant panel and 81.8% for food panel) among 3 MAST assays, implicating better specificity than other two assays (Table 5). Polycheck Allergy has been reported to have similar specificity (93.5%) with an RIDA Allergy Screen (90.0%) or MAST CLA (96.0%) [6]. In our study, ImmunoCAP assay was performed only on discrepant sera, which could make some different results from previous study [6].

For individual allergens, on House dust, which is most common allergen in Korean population [7] and on Peach, which showed most common discrepant results in our study, better sensitivities of MAST Optigen were suspected (Table 4). From previous study, when compared to allergen skin test, MAST CLA showed best performance on D. farinae [6]. However, because of the retrospective design of our study, the small number of ImmunoCAP assay results due to shortage of residual sera is a limitation to see the performance of MAST assays on individual allergens. Further studies are needed on larger number of samples to compare the performance of MAST assays on individual allergens. 

## 5. Conclusions

The 3 MAST assays: RIDA Allergy Screen, MAST Optigen, and Polycheck Allergy showed moderate agreements among them. In comparison with ImmunoCAP allergen-specific IgE test, MAST Optigen showed better agreement than other assays in the food panel. Better sensitivity of MAST Optigen and better specificity of Polycheck Allergy were suspected. Further studies are needed in larger number of samples to know the performance of MAST assays for individual allergens.

## Figures and Tables

**Table 1:** Comparison of allergens and classes of three MAST assays.

	RIDA Allergy Screen	MAST Optigen	Polycheck Allergy
Inhalant allergens included in common	Soy bean, Milk, Egg White, Crab, Shrimp, Peach, Acacia, Ash mix, Birch-alder mix, Sallow willow, Hazelnut, Cedar Japanese, Oak white, Sycamore mix, Bermuda grass, Orchard grass, Timothy grass, Rye Cultbatd, Goldenrod, Pigweed, Russian thistle, Dandelion, Mugwort, Ragweed short, Alternaria, Aspergillus, Cladosporium, Penicillium, Cat, Dog, Cockroach Mix, House dust, D. farinae, D. pteronyssinus		

Inhalant allergens included only in each reagent	Sweet vernal grass, Reed, Pine, Ox-eye-daisy	Cottonwood East	Redtop, Lilac, Fescue meadow, Latex, Tyrophagus putrescentiae, Ox-eye-daisy

Food allergens included in common	Soy beans, Milk, cheese, Egg white, Crab, Shrimp, Tuna, Codfish, Salmon, Pork, Chicken, Beef, Citrus mix, Wheat flour, Rice, Barley meal, Garlic, Peanut, Yeast bakers, Birch-Alder mix, Oak white, Rye, Mugwort, Ragweed short, Alternaria, Cat, Dog, Cockroach mix, House dust, D. farinae, D. pteronyssinus, Buckwheat meal		

Food allergens included only in each reagent	Aspergillus, Cladosporium, Onion, Acarus siro, Tomato, Candida albicans		Tomato, Timothy grass pollen, Cacao, Mackerel, Potato, Sweet chestnut

Class 0	0.0–0.34	0–26	0.0–0.34
1	0.35–0.69	27–65	0.35–0.69
2	0.7–3.49	66–142	0.7–3.49
3	3.5–17.49	143–242	3.5–17.49
4	17.5–49.99	>242	17.5–49.99
5	50.00–99.99		50.00–99.99
6	>100		>100
Unit	IU/mL	LUs	kU/L

**Table 2:** Concordance among three MAST assays.

		RIDA		Agreement (%)	kappa	Polycheck		Agreement (%)	kappa
		N*	P^†^			N	P		
Inhalant panel									
MAST Optigen	N	529	26	87.6	0.59	529	11	86.8	0.51
	P	61	84			79	61		
Polycheck	N	574	54	89.9	0.55				
	P	17	55						
Food panel									
MAST Optigen	N	600	23	91.3	0.60	552	32	90.6	0.61
	P	40	57			32	64		
Polycheck	N	572	31	88.3	0.46				
	P	51	46						

RIDA: RIDA Allergy Screen, Polycheck: Polycheck Allergy, N: negative, P: positive. *Number of tests with negative results was shown. ^†^Number of tests with positive results was shown.

**Table 3:** Antigen panels with discrepant results among three MAST assays.

RIDA	MAST Optigen	Polycheck	Antigen panel*
Inhalant panel			

+	+	−	Rye cultbatd, Cockroach mix, Birch-alder mix, Orchard grass, Timothy grass, Goldenrod, Dandelion, Mugwort
+	−	−	Sycamore mix (4), Goldenrod
−	+	+	House dust (4), D. farinae
−	+	−	Peach (5), Pigweed (4), Mugwort (3), Dandelion, Cockroach Mix, Milk, Crab, Birch-alder mix,
−	−	+	Hazelnut
+	NT	−	Ox-eye-daisy (4)

Food panel			

+	+	−	Beef (3), Milk
+	−	−	Alternaria, Pork
−	+	+	Peanut, Soy beans, Birch-alder mix, Yeast bakers, Cat
−	+	−	Cheese
−	−	+	House dust (4)

RIDA: RIDA Allergy Screen, Polycheck: Polycheck Allergy. NT: not tested. *Antigen panels with ≥2 discrepant samples were shown. Number of samples was shown in parenthesis when it was ≥3.

**Table 4:** Concordance of three MAST assays with ImmunoCAP according to antigen panels.

	Agreement with ImmunoCAP(+)			Agreement with ImmunoCAP(−)		
	RIDA	MAST Optigen	Polycheck	RIDA	MAST Optigen	Polycheck
Inhalant panel*						
House dust	0/3 (0%)^†^	3/3 (100%)	3/3 (100%)	NA	NA	NA
Milk	NA	NA	NA	2/3 (66.7%)	0/3 (0%)	3/3 (100%)
Mugwort	1/1 (100%)	1/1 (100%)	0/1 (0%)	1/1 (100%)	0/1 (0%)	1/1 (100%)
Crab	1/1 (100%)	1/1 (100%)	0/1 (0%)	1/1 (100%)	0/1 (0%)	1/1 (100%)
Timothy grass	1/1 (100%)	1/1 (100%)	0/1 (0%)	0/1 (0%)	1/1 (100%)	1/1 (100%)
Dandelion	NA	NA	NA	0/1 (0%)	0/1 (0%)	1/1 (100%)
Peach	0/3 (0%)	3/3 (100%)	0/3 (0%)	1/1 (100%)	0/1 (0%)	1/1 (100%)
Cockroach mix	NA	NA	NA	1/1 (100%)	0/1 (0%)	1/1 (100%)
Birch-alder mix	NA	NA	NA	1/2 (50%)	0/2 (0%)	2/2 (100%)
Hazelnut	NA	NA	NA	1/1 (100%)	1/1 (100%)	0/1 (0%)
Alternaria	1/1 (100%)	0/1 (0%)	1/1 (100%)	0/1 (0%)	1/1 (100%)	1/1 (100%)
Rye, cultbatd	1/1 (100%)	1/1 (100%)	0/1 (0%)	NA	NA	NA
Aspergillus	NA	NA	NA	0/1 (0%)	1/1 (100%)	1/1 (100%)
Sycamore mix	NA	NA	NA	0/1 (0%)	1/1 (100%)	1/1 (100%)
Cedar Japanese	NA	NA	NA	0/1 (0%)	1/1 (100%)	1/1 (100%)
Ox-eye-daisy	1/1 (100%)	NA	0/1 (0%)	NA	NA	NA
Orchard grass	1/1 (100%)	1/1 (100%)	0/1 (0%)	NA	NA	NA
Food panel*						
D. pteronyssinus	0/1 (0%)	1/1 (100%)	1/1 (100%)	NA	NA	NA
House dust	NA	NA	NA	2/2 (100%)	2/2 (100%)	0/2 (0%)
Milk	1/3 (33.3%)	3/3 (100%)	1/3 (33.3%)	0/1 (0%)	0/1 (0%)	1/1 (100%)
Mugwort	NA	NA	NA	0/1 (0%)	1/1 (100%)	1/1 (100%)
Dog	1/1 (100%)	1/1 (100%)	0/1 (0%)	0/1 (0%)	1/1 (100%)	1/1 (100%)
Egg white	0/2 (0%)	2/2 (100%)	1/2 (50%)	NA	NA	NA
Soy beans	0/1 (0%)	1/1 (100%)	1/1 (100%)	NA	NA	NA
Shrimp	0/1 (0%)	1/1 (100%)	0/1 (0%)	NA	NA	NA
cheese	1/3 (33.3%)	3/3 (100%)	0/3 (0%)	0/1 (0%)	1/1 (100%)	1/1 (100%)
Garlic	1/1 (100%)	0/1 (0%)	0/1 (0%)	NA	NA	NA
Alternaria	1/2 (50%)	1/2 (50%)	1/2 (50%)	0/1 (0%)	1/1 (100%)	1/1 (100%)
Cat	0/1 (0%)	1/1 (100%)	1/1 (100%)	NA	NA	NA
Codfish	1/1 (100%)	1/1 (100%)	0/1 (0%)	NA	NA	NA
Salmon	1/2 (50%)	2/2 (100%)	0/2 (0%)	NA	NA	NA
Pork	NA	NA	NA	0/2 (0%)	2/2 (100%)	2/2 (100%)
Chicken	1/2 (50%)	1/2 (50%)	0/2 (0%)	NA	NA	NA
Beef	NA	NA	NA	0/2 (0%)	0/2 (0%)	2/2 (100%)
Citrus mix	1/1 (100%)	1/1 (100%)	0/1 (0%)	NA	NA	NA

RIDA: RIDA Allergy Screen, Polycheck: Polycheck Allergy, NA: not available. *Antigen panels were shown in the order of decreasing positive rate in Korean population [7]. ^†^Number of positive results/number tested (%).

**Table 5:** Concordance of three MAST assays with ImmunoCAP on discrepant sera among three MAST assays.

		ImmunoCAP		Agreement (%)		
		N*	P^†^	Total (kappa)	ImmunoCAP(+)	ImmunoCAP(−)
Inhalant panel						
RIDA	N	8	6	51.7 (0.04)	53.8	50.0
	P	8	7			
MAST Optigen	N	6	1	60.7 (0.27)	91.7	37.5
	P	10	11			
Polycheck	N	15	9	65.5 (0.26)	30.8	93.8
	P	1	4			
Food panel						
RIDA	N	2	13	33.3 (−0.38)	40.9	18.2
	P	9	9			
MAST Optigen	N	8	3	81.8 (0.59)	86.4	72.7
	P	3	19			
Polycheck	N	9	16	45.5 (0.07)	27.3	81.8
	P	2	6			

RIDA: RIDA Allergy Screen, Polycheck: Polycheck Allergy, N: negative, P: positive. *Number of tests with negative results was shown. ^†^Number of tests with positive results was shown.
